# Research on Stability of Removal Function in Figuring Process of Mandrel of X-Ray-Focusing Mirror with Variable Curvature

**DOI:** 10.3390/mi15121415

**Published:** 2024-11-25

**Authors:** Jiadai Xue, Yuhao Li, Mingyang Gao, Dongyun Gu, Yanlin Wu, Yanwen Liu, Yuxin Fan, Peng Zheng, Wentao Chen, Zhigao Chen, Zheng Qiao, Yuan Jin, Fei Ding, Yangong Wu, Bo Wang

**Affiliations:** 1 Center for Precision Engineering, Harbin Institute of Technology, Harbin 150001, China; 15765517664@163.com (J.X.);; 2 School of Mechanical and Electrical Engineering, China University of Mining and Technology-Beijing, Beijing 100083, China; 3 College of Mechanical and Electrical Engineering, Northeast Forestry University, Harbin 150040, China

**Keywords:** variable-curvature mandrel, compliant figuring process, the mid-spatial frequency error, real-time correction of TIFs

## Abstract

Over the past 30 years, researchers have developed X-ray-focusing telescopes by employing the principle of total reflection in thin metal films. The Wolter-I focusing mirror with variable-curvature surfaces demands high precision. However, there has been limited investigation into the removal mechanisms for variable-curvature X-ray mandrels, which are crucial for achieving the desired surface roughness and form accuracy, especially in reducing mid-spatial frequency (MSF) errors. It is essential to incorporate flexible control in deterministic small-tool polishing to improve the tool’s adaptability to curvature variations and achieve stable, Gaussian-like tool influence functions (TIFs). In this paper, we introduce a curvature-adaptive prediction model for compliance figuring, based on the Preston hypothesis, using a compliant shaping tool with high slurry absorption and retention capabilities. This model predicts the compliance figuring process of variable-curvature symmetrical mandrels for X-ray grazing incidence mirrors by utilizing planar tool influence functions. Initially, a variable-curvature pressure model was developed to account for the parabolic and hyperbolic optical surfaces’ curvature characteristics. By introducing time-varying removal functions for material removal, the model establishes a variable-curvature factor function, which correlates actual downward pressure with parameters such as contact radius and contact angle, thus linking the variable-curvature surface with a planar reference. Subsequently, through analysis of the residence time distribution across different TIF models, hierarchical filtering, and PSD distribution, real-time correction of the TIFs was achieved to enable customized variable-curvature polishing. Furthermore, by applying a time-varying deconvolution algorithm, multiple rounds of flexible polishing iterations were conducted on the mandrels of a rotationally symmetric variable-curvature optical component, and the experimental results demonstrate a significant improvement in form accuracy, surface quality, and the optical performance of the mirror.

## 1. Introduction

The development of freeform optics in symmetric optical systems presents significant challenges for ultra-precision optics manufacturing, particularly in compensating for the constraints posed by unconventional optical shapes, such as complex large telescope mirrors [1] and freeform optics, like a micro-lens array [2,3], to mention a few. Specifically, the advancement of large-area X-ray focusing imaging technology has catalyzed the rapid development of X-ray astronomy over recent decades. These advanced imaging systems have been widely implemented in space missions by the United States, Italy, Germany, Russia, and other countries. Notable examples include the BeppoSAX for ASI [4], XMM-Newton for ESA [5], JET-X/SWIFT for NASA [6], eROSITA for MPE [7], IXPE for NASA [8], and Einstein Probe for CAS-ESA [9]. Most current and upcoming X-ray focusing telescopes in orbit utilize Wolter-type optics, originally designed by German physicist Hans Wolter in 1952. These telescopes meet the Abbe sine condition of grazing incidence, with Wolter I, II, and III types featuring various combinations of paraboloid, hyperboloid, and ellipsoid shapes [10]. Moreover, the nickel electroforming replication technique facilitates the production of thin, lightweight mirrors suited for Wolter-type optics [11]. The imaging quality of these mirrors largely depends on the shape accuracy of the mandrels, which serve as rigid cylindrical substrates made from materials such as quartz glass molds [12], electroless nickel-coated aluminum [13], or fused silica [14]. Fabricating mandrels with variable curvature poses significant challenges, as achieving the desired surface roughness of 0.5 nm RMS or lower and a form accuracy of less than 200 nm P-V is critical for reducing mid-spatial frequency errors.

The machining accuracy of mandrels is directly linked to the mirror’s focusing quality. The optical performance of focusing mirrors is influenced not only by shape error but also by mid- and high-spatial frequency errors. Variable-curvature figuring is an advanced process in optics manufacturing used to precisely shape the surfaces of X-ray mandrels. Fluid-based figuring utilizes a polishing slurry or other fluid to incrementally remove material, thereby achieving the desired surface profile. This method relies on principles of fluid dynamics and controlled material removal to fine-tune the curvature and figure of optical surfaces. Transitioning from traditional, manually uncertain surface technologies to controlled compliant small-tool surface technology—facilitated by computer-controlled optical surfaces (CCOSs)—enables more consistent removal functions. In contrast, traditional X-ray axial figuring processes, which use rigid tools and random figuring, are less effective at reducing axial slope errors, especially for surfaces with significant curvature (e.g., large diameters and short focal lengths). Vernani [12] outlined the specifications for X-ray mandrels and contributed to advancing figuring and polishing methods at Italy’s Media Lario company. Media Lario employs the IRP 1200X (Zeeko Ltd., Leicestershire, United Kingdom), a bridge-based, seven-axis CNC optical polishing and figuring machine, which offers a stable removal rate, eliminates diamond turning “record groove” marks, and enhances form correction and texture. This approach uses a sub-diameter physical tool combined with a polishing slurry. The tool’s membrane, shaped like a spherical bonnet, conforms closely to the mandrel’s surface, ensuring effective contact on aspheric surfaces. As a result, corrective figuring can significantly improve axial slope errors, reducing an initial PtV error of 500 nm to 60 nm after diamond turning.

Kilaru [13] detailed the development initiatives at NASA’s Marshall Space Flight Center (MSFC), which focused on two approaches: direct fabrication of mirror shells—using diamond turning and deterministic computer-controlled polishing (Zeeko IRP 600, Leicestershire, United Kingdom)—and the replication of mirror shells from polished mandrels. Corrections for a wide range of figure error spatial frequencies can be achieved by adjusting the material removal rate, which depends on parameters such as the tool feed rate, bonnet pressure, spindle rotation rate, tool offset, precession angle, and bonnet angle. Additional factors include the bonnet characteristics (radius, thickness, and elastic modulus) and slurry properties (particle grit size and solids fraction). These adjustments enable the development and refinement of algorithms to apply the wear function for precise surface error correction. The latest applications on NiP-coated, vertically oriented cylindrical mandrels have achieved grazing-incidence specifications with slope errors within 0.5 arc seconds, yielding a half-power diameter (HPD) of less than 2 arc seconds for spatial wavelength ranges of 7 mm or more (a limitation of the current tooling). While both of the aforementioned figuring processes are capable of precisely finishing X-ray mandrels, they require extensive experimentation and a significant time investment. There remains a need for deeper investigation into the removal mechanisms for variable-curvature X-ray mandrels to consistently achieve the desired surface roughness and form accuracy.

Further analysis illustrates the key point that non-linearity exists for the removal rate, which affects the convergence rate in the X-ray mandrel figuring process because of variable curvature, causing non-linearity of the tool influence function (TIF). Developed deconvolution methods allow the dwell time map for the given surface error and TIF to be solved with high accuracy. The rate at which convergence occurs is a key metric for assessing the effectiveness of the computational procedure, and it is highly dependent on the real-time correction of TIFs. TIFs are significantly affected by the degree of conformance between the forms of the polishing pad and the workpiece. Many scholars have investigated the removal mechanism based on the renowned Preston hypothesis [14], which predicts the material removal rate (MRR) combined with the contact mechanics model. Schinhaerl et al. [15] studied TIFs with customized dwell times for various sub-regions to accomplish effective iterative figuring without additional mid-spatial frequency (MSF) and high-spatial frequency (HSF) errors under the maximum feed rate. Wan et al. [16] proposed a new analytical TIF model as a function associated with the relative curvature difference for sub-aperture polishing when there is form deviation between the polishing pad and the workpiece. Ren et al. [17] established a material removal model using the Hertz contact theory to investigate the effects of the polishing parameters on the deviation characteristics of the material removal profile. A wear index for polishing in relation to the material removal was investigated for the polishing conditions based on the Archard wear equation, and an approach was developed for calculating the material removal profile of polishing using a soft tool with fixed abrasives [18]. However, the stability of the removal function for variable curvatures in the figuring process, especially for the mandrel of X-ray-focusing mirrors, has not been reported in publication.

In this paper, we demonstrate a curvature-adaptive prediction model for figuring based on the Preston hypothesis, employing a compliance shaping tool with high slurry absorption and retention capacity. This model effectively predicts the compliance figuring process for variable-curvature symmetrical mandrels, specifically designed for X-ray grazing incidence mirrors, using planar tool influence functions (TIFs). Initially, a variable-curvature pressure model was developed based on the variable-curvature characteristics of parabolic and hyperbolic optical surfaces, alongside the introduction of time-varying removal functions for material extraction. This model establishes a variable-curvature factor function by correlating actual downward pressure with key parameters, such as contact radius and contact angle, thereby linking the variable-curvature surface with a planar reference. Subsequently, based on the residence time distribution across different TIF models, hierarchical filtering, and power spectral density (PSD) distribution, the variable-curvature surface and planar surface guide each other to enable real-time TIF correction, achieving customized variable-curvature polishing. Furthermore, using a time-varying deconvolution algorithm, multiple rounds of flexible polishing iterations are applied to the mandrels of focusing mirrors—rotationally symmetric variable-curvature optical components. The experimental results reveal substantial improvements, with form accuracy for the parabolic surface reducing from 1157 nm PV to 234 nm, and for the hyperbolic surface, from 274 nm PV to 105 nm, while maintaining surface roughness below 2 nm rms, effectively reducing mid-spatial frequency ripples.

## 2. The Removal Mechanism and Characteristics of the Compliant Figuring Process

### 2.1. The Variable-Curvature Mandrel with Parabolic and Hyperbolic Optical Surfaces

**Figure 1:**
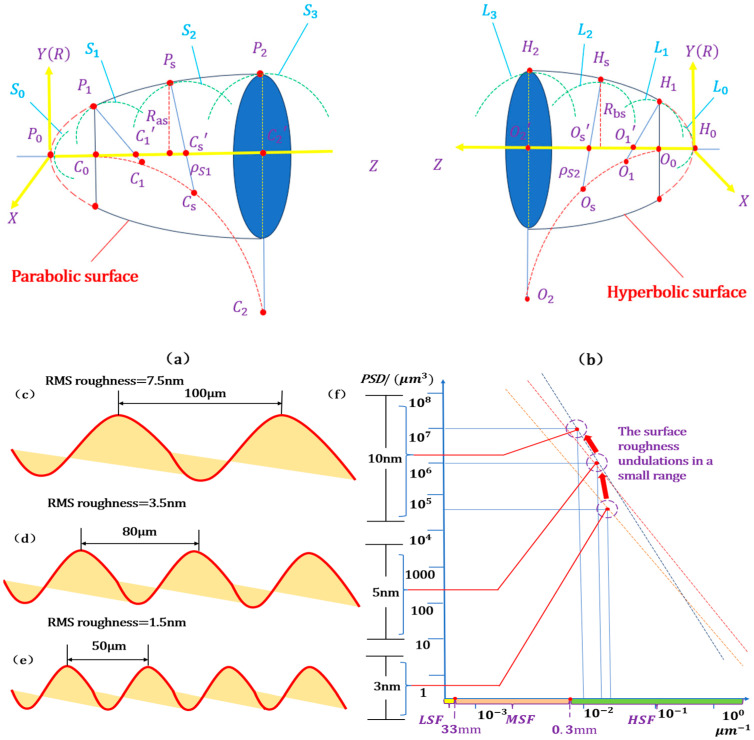
Optical characteristics of mandrel with variable curvature: (**a**,**b**) Geometric analysis of hyperboloid and paraboloid of mandrel; (**c**–**e**) spatial wavelength of different sizes; (**f**) influence of spatial wavelength amplitude change on PSD curve.

Controlling the form accuracy and surface roughness of high-precision replication mandrels is crucial for achieving the highest precision in manufacturing focusing mirrors with high angular resolution and large effective areas, using electroformed nickel-replicated (ENR) technology. This minimizes splicing errors while maintaining high production efficiency. The mandrel’s surface consists of hyperbolic and parabolic shapes, as shown in Figure 1a,b, with a distinctive, variable-curvature, axisymmetric, full-diameter freeform. This is generated through an electroless nickel–phosphorus alloy and single-point diamond turning (SPDT). According to the parabolic surface shown in Figure 1a, $P_{0}$ is the origin of the parabola, $P_{1}$ and $P_{2}$, respectively, are the initial and terminal points of the parabola part of the mandrel, $C_{1}$ and $C_{2}$ are the corresponding centers of curvature, and $S_{1}$ and $S_{3}$ are the curvature arc. With the gradual increase in the radius of gyration $R_{as}$ of the parabolic surface, the radius of curvature $\rho_{s_{1}}$ increases, ultimately forming the paraboloid curvature center trajectory $C_{1}C_{s}C_{2}$, which is the comparable geometric relationship of the hyperbolic surface shown in Figure 1b.

However, the mandrel manufacturing errors, including deflection vibration, pitch vibration, and the influence of the thermal expansion coefficient, result in a considerable error in the surface accuracy of the forming die (the surface error from two hundred nm to one thousand nm). Hence, it is necessary to make substantial corrections (hundred-nanometer level) to the surface accuracy and ensure that the surface roughness inductions are within a small range (6–7 nm) while reducing the medium-spatial frequency ripple error, i.e., preserving the high-spatial frequency (HSF) and lowering the low-spatial frequency (LSF) and mid-spatial frequency (MSF) error. To accurately determine the distribution of mandrel surface errors, a precise division of spatial frequency should be conducted. Power spectral density (PSD) is a crucial means of evaluating surface errors that vary with spatial frequencies. The error variation of the freeform at different spatial frequencies can be described by multiple 1-D or a 2-D PSD. To effectively determine the underlying cause of roughness variations on a surface, it is imperative to restrict the PSD curve by concentrating on two distinct spatial frequencies within a designated region. The MSF pertains to the errors in the spatial wavelength band between 0.3 mm and 33 mm. The LSF pertains to errors at wavelengths greater than 33 mm, while the HSF pertains to errors at wavelengths less than 0.3 mm. As presented in Figure 1c–f, the three frequency band errors are due to high-frequency errors. The area covered by the 1-D PSD curve gradually increases, and the slope decreases as the surface roughness worsens due to the multiple iterations of the removal kernels. As the spatial period increases, the undulations grow in magnitude, leading to slight increases in surface roughness within a narrow range. Hence, it is easier to achieve ultra-smooth polishing by utilizing a stable tool with a large full width at half maximum (FWHM), uniformly reducing surface roughness to the sub-nanometer level while maintaining high form accuracy.

### 2.2. The Material Removal Mechanism of the Compliant Figuring Process

The ultra-precision SPDT achieves high form accuracy and low surface roughness for a single-crystal diamond with a nanoscale radius. However, the optical surface following SPDT leaves a periodic mid-spatial frequency error as a result of temperature control and the misfit between the vibration of the single-crystal diamond tool and the ultra-precision machine during the precision cutting operation. These errors are commonly assumed as diamond turning marks. Small-angle scattering occurs, reducing the peaks of the PSF. The performance of an X-ray optical system is affected by this type of spatial frequency error, causing image factor degradation. Low-frequency errors arise from long wavelength surface undulations, resulting in spatial wavefront aberrations, such as spherical aberration and coma.

**Figure 2:**
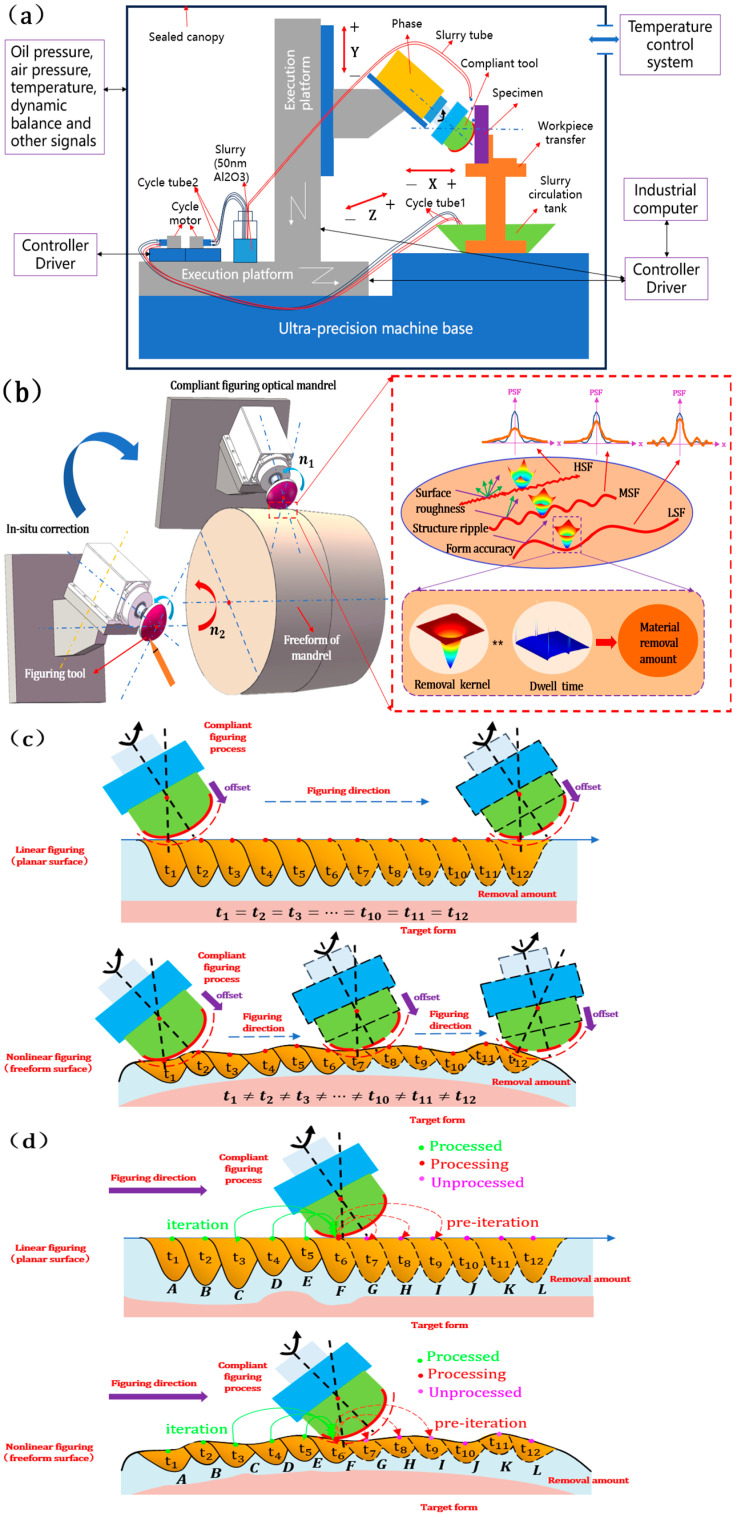
Variable-curvature optical surface polishing mechanism: (**a**) Variable-curvature polishing system; (**b**) variable-curvature polishing principle; (**c**) stable removal function for material removal; (**d**) time-varying removal function for material removal.

A semi-rigid trimming tool constructed of a rigid substrate and a flexible figuring head is employed to achieve customized flexible figuring for a freeform surface with variable curvature. Regulating the integrated curvature radius $\rho_{\Sigma }$ and offset $d_{t_{e}}$ generates suitable maximum removal depth (MRD) and FWHM, and corresponding spatial wavelengths are restricted to remove the expected spatial errors with a depth of hundreds of nanometers and a spatial wavelength of tens of millimeters, as well as a wide range of low-frequency errors to ensure form accuracy. Despite the reduction in MSF, urgent issues still remain to be addressed, including the high-frequency error of small-scope spring backing and the mid-frequency error introduced by the tool itself. Hence, for the combined process of SPDT, CFP, and stress lapping polishing (SLP), a stable, wide FWHM for ultra-precision optical machining is imperative, removing the residual MSF and HSF and realizing the full-frequency domain of the error control. The research object of this paper was CFP with a corrective polishing-like process: the self-developed ultra-smooth figuring system, consisting of the CNC motion system, the compliant figuring system, and the temperature control system, and an in situ corrected figuring tool could realize customized flexible figuring for the planar and variable-curvature surface of optics, obtaining a stable and effective removal function and enhancing PSF with good imaging performance, as shown in Figure 2a,b. Based on the convolution (*) between and material removal kernel and the residence time distribution, the material amount could be obtained in deterministic compliant figuring.

Selecting an appropriate TIF, convolved with the corresponding residence time distribution, at a specific location on the optical surface is crucial to accounting for various spatial wavelength errors, generating the precise volume removal amount per unit of time. Theoretically, the tool head of the planar figuring traverses the entire optical surface in a predetermined path while maintaining a consistent feed rate, addressing spatial errors within the same frequency band with a uniform distribution of dwell time. Hence, the CFP generates a consistent TIF at each dwell point to effectively remove diamond turning (DT) marks introduced by SPDT to realize the form-preserving figuring. For the freeform surface figuring, with the varying undulation of the optical surface and the integrated curvature radius, the residence time distribution of the spatial error is uneven for the same spatial wavelength, generating a time-varying TIF at each dwell point and reducing the surface structure error for uniform material removal for the surface profiles with considerable range accuracy errors (from the hundred-nanometer level to thousand-nanometer level PV), as shown in Figure 2c.

However, the actual manufacturing situation is different from the aforementioned nominal forward figuring, based on the inverse convolution between the determined initial error and material removal kernel, obtaining the residence time distribution and achieving the deterministic compliant figuring with precise control of feed speed, according to the different spatial wavelengths. This type of manufacturing is reverse figuring, which produces an iterative error effect and includes the tool weariness, mismatch between the tool and the optical surface, and stability of the slurry. Firstly, as shown in Figure 2d, the pre-iteration has an impact on the unprocessed points G to I when the figuring tool is located at F. Subsequently, when the tool moves from points G to I, a family of pre-removal kernels formed by the pre-iterations and the residence time at each position cooperatively determine the actual TIF, generating an uneven volume removal rate. Moreover, when processed at point F, the TIF is simultaneously affected by the pre-iterations generated during the residence time at each of the processed points C to E. To comprehensively understand and effectively address the pre-iterative problem, it is imperative to investigate this from the perspective of path planning (including zigzag, Archimedes spiral, and pseudo-random) and iterative algorithms. When the raster path is utilized, the TIFs generate a whole-region iterative effect owing to the multiple-time one-dimensional overlapping marks of the figuring head with uniform dwell time distribution at each point and realizing stable material removal. When utilizing the Archimedes spiral path, the TIFs generate a local iterative effect owing to the density inhomogeneity of equivalent residence time distribution with the figuring head closing the optical element center, realizing sparse to dense iterative distributions and uneven material removal in the immediate vicinity. When a pseudo-randomness path is executed, the overlapping feature of the aforementioned unicursal pathway is avoided due to random distribution over the entire optical surface and observation of the never-intersect principle, suppressing the characteristic PSD peak and improving the imaging quality of the optics system.

Meanwhile, continuous motion with various surface velocities is achieved, and the near-neighbor iteration effect is revealed by iterative algorithms (including the convolutional trial algorithm, Lucy–Richardson deconvolutional algorithm, Weiner deconvolutional algorithm, and regularization algorithm) and prescribed pathways in combination with figuring shape errors composed of various spatial wavelengths. Therefore, it is critical to execute a comprehensive investigation on the impact of optical surfaces with different curvature characteristics on the TIF by adjusting the essential geometric parameters, including contact radius, edge contact angle, and misfit deformation, to compensate for the real-time offset and maintain the robustness and self-adaptivity of the TIF.

**Figure 3:**
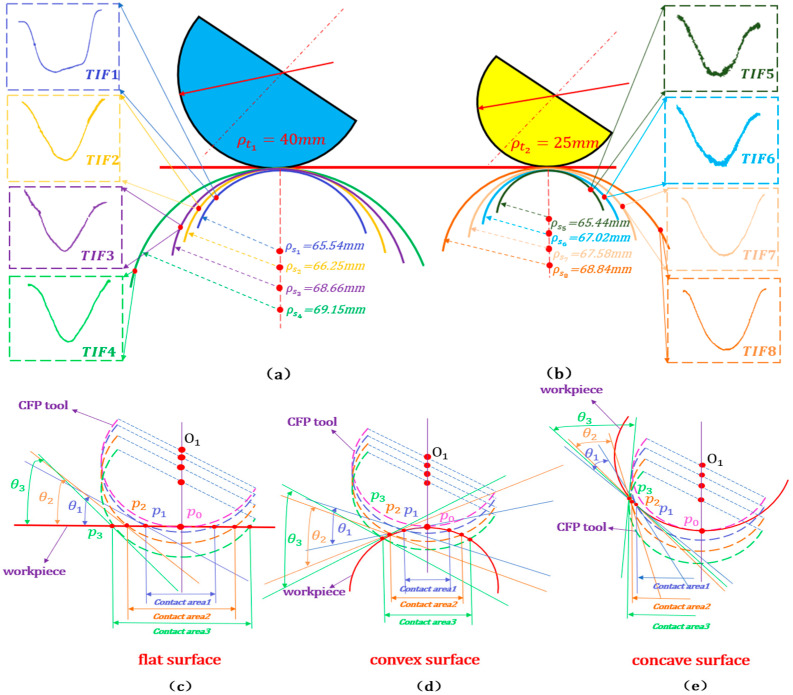
Matching flexible tools with variable curvature: (**a**,**b**) TIF; (**c**–**e**) performance of polishing tools on planar, convex, and concave workpieces with large and small radii of curvature matched with different radii of curvature under different pressures.

In order to verify the influence of the comprehensive radius of curvature on the TIF, the surface of the workpiece with a radius of curvature from 65.44 mm to 69.15 mm is selected with a radius of curvature as 25 mm and 40 mm, respectively. As shown in Figure 3a,b, simulation experiments were carried out on the surfaces of varying radii of curvature, utilizing the figuring tool with a specific radius curvature, generating a higher removal resolution, a lower removal efficiency, a larger FWHM of the TIF, and a lower Gaussian-type convergence rate with the same radius of curvature for the workpiece and increasing the radius of curvature of the tool. In contrast, the stability of material removal by the TIF and the Gaussian-type convergence rate were stably improved with the same tool curvature radius and increased workpiece curvature radius, and the material removal rate and FWHM of the TIF became proportionally larger, belonging to the Gaussian-like type for the trimming tool with a small curvature radius. With the increase in offset and pressure for the flexible figuring tool, the contact area and the edge contact angle increased, whether the figured specimen surface was flat, convex, or concave, as shown in Figure 3c–e. To accurately determine the impact of workpiece curvature on the TIF by adjusting the crucial geometric relationship, it is essential to establish a pressure model encompassing adaptable variable-curvature characteristics.

### 2.3. Motion Model and Analysis of CFP Characteristics

For small-tool figuring and polishing, e.g., bonnet polishing (BP), magnetorheological finishing (MRF), and fluid jet polishing (FJP), the TIF is of importance in investigating the removal mechanism accurately. Because the expected material removal is obtained by the convolution of the TIF and the dwell time, the Preston equation is as follows:

(1) $$MRR=\frac{∆z}{∆t}=K\left(k_{1},k_{2},k_{3},k_{4}\right)\cdot {p\left(x,y\right)}^{a}\cdot {v\left(x,y\right)}^{b}$$

where MRR is the material removal rate; ∆z is the amount of removal material by the figuring tool; K is the Preston coefficient considering $k_{1}\;$ as the workpiece material, $k_{2}\;$ as the abrasive of slurry, $k_{3\;}\;$ as the flexible figuring tool material, and $k_{4}\;$ as the other factors; $p\left(x,y\right)\;$ is the contact pressure on the workpiece at point $\left(x,y\right)$; $v\left(x,y\right)\;$ is the relative velocity between the workpiece and tool at point $\left(x,y\right)$; a is the coefficient of $p\left(x,y\right)$; b is the coefficient of $v\left(x,y\right)$; and x and y are the coordinate axes established with the center of the contact area as the origin and the distances from the center on the *x*-axis and *y*-axis, respectively. The Preston equation is influenced by various factors, such as the type and size of abrasive particles in the polishing solution, the solution’s concentration, and the operating temperature, and cannot precisely define the relationship between all the figuring parameters and material removal. Therefore, it becomes necessary to simplify the equation, allowing for a more focused quantification of the Preston coefficient, with emphasis on velocity and pressure.

#### The Curvature Contact Stress Model and Calculation

**Figure 4:**
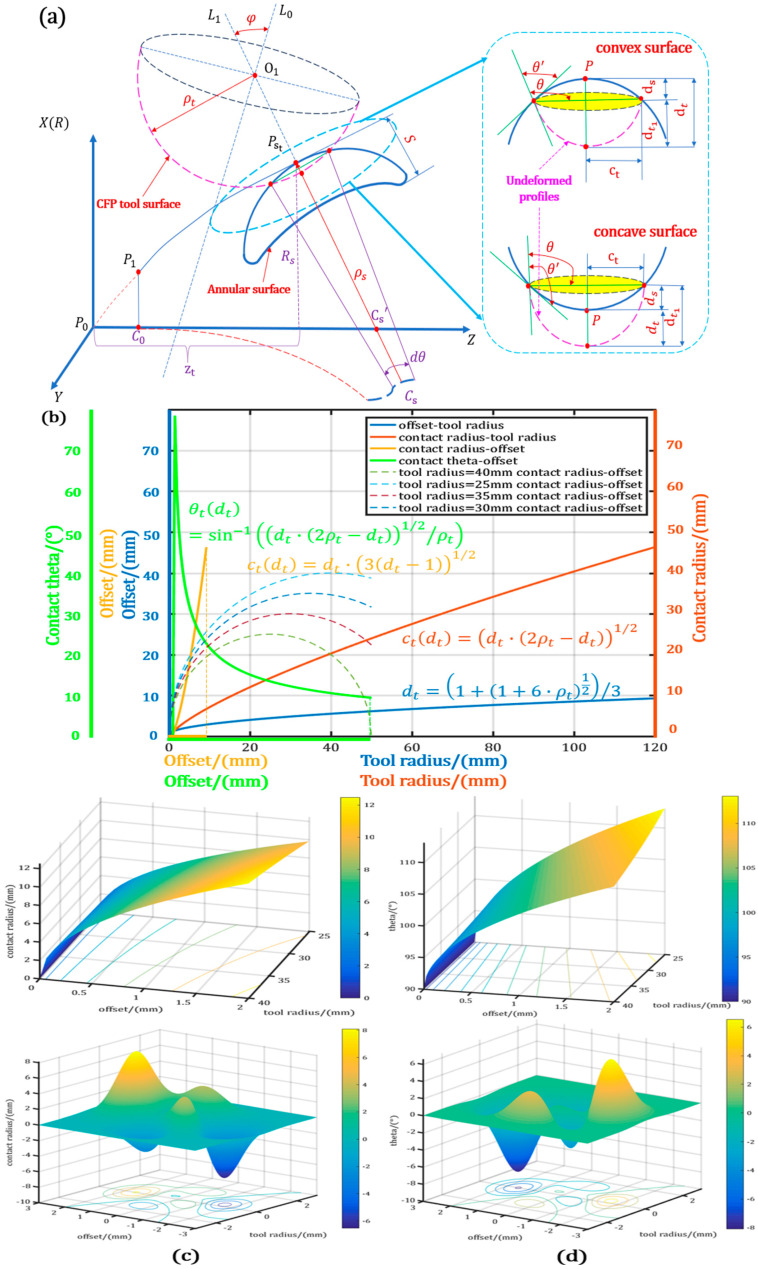
Pressure model with variable curvature: (**a**) Schematic diagram of pressure distribution of convex and concave workpieces; (**b**) variation in key parameters of plane pressure under limit conditions; (**c**,**d**) influence of distribution of plane pressure and tool curvature radius on contact radius and edge contact angle.

A variable-curvature contact pressure model was established based on a dual-curvature spherical contact geometric model, including the flexible sphere of the figuring tool and the rigid sphere of variable-curvature optics, as shown in the schematic diagram of Figure 4a. The flexible polishing tool with the radius of curvature $\rho_{t}$ is selected to traverse from $P_{1}$ to $P_{S_{t}}$, where it is in contact with the workpiece, and the corresponding radius of curvature is $\rho_{s}$, the radius of rotation is $R_{s}$, and the center of curvature is *C_s_*. The sag of the local torus formed in the contact area between the tool and the workpiece is S at the position $z_{t}$ in the Z-axis, the rotation angle of the contact area is dθ, the edge contact angle is θ, and the tool rotation centerline is $L_{0}$. The tool’s active polishing centerline is $L_{1}$, the angle between $L_{1}$ and $L_{0}$ is the precession angle φ, and the vertical shape variable $d_{t}$ is larger when the workpiece is convex.

According to the Hertzian contact theorem and the characteristics of CFP, the expression of relative pressure and compressive stress distribution can be given as follows:

(2) $$\sigma_{t}\left(x_{t},y_{t}\right)=\frac{P_{t}\left(x_{t},y_{t}\right)}{S_{\Omega_{t}}}=\frac{P_{t}\cdot {\left(1-\frac{{x_{t}}^{2}}{{c_{t}}^{2}}-\frac{{y_{t}}^{2}}{{c_{t}}^{2}}\right)}^{1/2}}{\pi \cdot c_{t}\cdot c_{t}}=\frac{P_{t}{\left({c_{t}}^{2}-{x_{t}}^{2}-{y_{t}}^{2}\right)}^{1/2}}{\pi {c_{t}}^{3}}$$

where $P_{t}$ is the pressure accumulated at a certain moment executing on the tool within the dwell time t, corresponding to the workpiece curvature $\rho_{s}$ with the unit of $P_{a}$; $S_{\Omega_{t}}$ is the area of the contact region corresponding to $P_{t}$, which sets the dimension unit as $m^{2}$; and $c_{t}$ is the distance between the regional center of $S_{\Omega_{t}}$ and the contact boundary, which sets the dimension unit as m.

The relationship between the contact radius $c_{t}$ and the overlap form variable d is established as follows:

(3) $$c_{t}\left(d_{t}\right)={\left(\left(-2{d_{t}\rho }_{s}+{d_{t}}^{2}\right)\cdot \left(-2\rho_{t}+d_{t}\right)\cdot \left(2\rho_{s}+2\rho_{t}-d_{t}\right)\right)}^{1/2}/2\left(\rho_{s}+\rho_{t}-d_{t}\right)$$

where $d_{t}$ is the overlap deformation variable of the flexible tool composed with the initial deformation and the offset deformation, $\rho_{s_{t}}$ is the curvature of freeform workpiece, $\rho_{t}$ is the curvature of CFP tool, and the angle of the contact margin $\theta^{\prime }$ can be described as follows:

(4) $$\theta_{t}\left(d_{t}\right)=\sin^{-1}\left(c_{t}/\rho_{t}\right)+\cos^{-1}\left(c_{t}/\rho_{s}\cdot \left(\tan\left(\sin^{-1}(c_{t}/\rho_{s})\right)\right)\right)$$

Hence, $d_{t}$ has a relationship between $c_{t}$ and ${\theta_{t}}^{\prime }$, which can directly investigate inherent contact deformation. According to the geometric formulas, the corresponding radii of rotational symmetry are hyperboloid and paraboloid, respectively, and can be calculated as follows:

(5) $$R_{s_{p}}={\rho_{z}}^{2}\cdot \sum_{i=0}^{1}a_{i}\cdot {\left(\left(z_{t}-z_{0}\right)/\rho_{z}\right)}^{i}$$

(6) $$R_{s_{h}}={\rho_{z}}^{2}\cdot \sum_{i=0}^{2}b_{i}\cdot {\left(\left(z_{t}-z_{0}\right)/\rho_{z}\right)}^{i}$$

where $z_{t}$ is the current polishing position, $z_{0}$ is the middle position of the workpiece with variable curvature, $\rho_{z}$ is the curvature of freeform workpiece, and $\rho_{t}$ is the curvature of the figuring tool with $a_{i}$ and $b_{i}$ as the geometric constants of the surface.

From the deformation perspective, the figuring tool generates the initial misfit deformation $\delta_{0}$ with the workpiece surface under no load. Subsequently, the tool and specimen, respectively, produce deformation $\delta_{s}$ and $\delta_{t}$, contributing to the equivalent curvature $\rho_{\Sigma }$, which can be expressed by $\frac{1}{\rho_{\Sigma }}=\frac{1}{\rho_{s}}+\frac{1}{\rho_{t}}$, and the difference in the elastic properties, and form the contact zone with the radius of $c_{t}$. Nevertheless, the curvature specimen is totally rigid, and $\delta_{s}=0$. As a result, the total deformation $d_{t}$ can be expressed by

(7) $$d_{t}=\delta_{s}+\delta_{t}+\delta_{0}=d_{t_{e}}+{\Delta \delta \left(d_{t}\right)}^{*}$$

where $\delta_{t}$ is composed of the effect offset $d_{t_{e}}\;$ of the figuring tool and the offset misfit deformation $\Delta \delta (c_{t})$, which can take $\delta_{0}$ together as the equivalent misfit deformation ${\Delta \delta \left(d_{t}\right)}^{*}$, described as follows:

(8) $${\Delta \delta \left(d_{t}\right)}^{*}=\frac{{c_{t}(d_{t})}^{2}+{c_{0}}^{2}}{2\rho_{\Sigma }}$$

The total deformation $d_{t}$ can be obtained by Formulas (1)–(7):

(9) $$\begin{array}{c} {d_{t}}^{4}/{\rho_{0}}^{2}+4{d_{t}}^{3}\left(2\rho_{\Sigma }/\rho_{0}-1\right)+4{d_{t}}^{2}\left(1+\rho_{t}\rho_{s_{t}}/{\rho_{0}}^{2}-4\rho_{\Sigma }/\rho_{0}-2\rho_{\Sigma }c_{e}/{\rho_{0}}^{2}\right)\\ +8d_{t}\left(\rho_{\Sigma }+2\rho_{\Sigma }c_{e}/\rho_{0}-\rho_{t}\rho_{s_{t}}/\rho_{0}\right)-8\rho_{\Sigma }c_{e}=0 \end{array}$$

When the processing optical surface is flat, $\rho_{s_{t}}=+\infty$ can be obtained between total deformation $d_{t}$ with tool radius of curvature $\rho_{t}$, edge contact angle $\theta_{t}$ and contact radius $c_{t}$. The relationship between a free curved surface and plane, which is essential for surfaces of varying curvature in CFP, is established as follows:

(10) $$d_{t}=\left(1+{\left(1+6\cdot \rho_{t}\right)}^{\frac{1}{2}}\right)/3$$

(11) $$\theta_{t}\left(d_{t}\right)=\sin^{-1}\left(\frac{{\left(d_{t}\cdot \left({2\cdot \rho }_{t}-d_{t}\right)\right)}^{\frac{1}{2}}}{\rho_{t}}\right)$$

(12) $$c_{t}\left(d_{t}\right)=d_{t}\cdot {\left(3\left(d_{t}-1\right)\right)}^{1/2}$$

**Figure 5:**
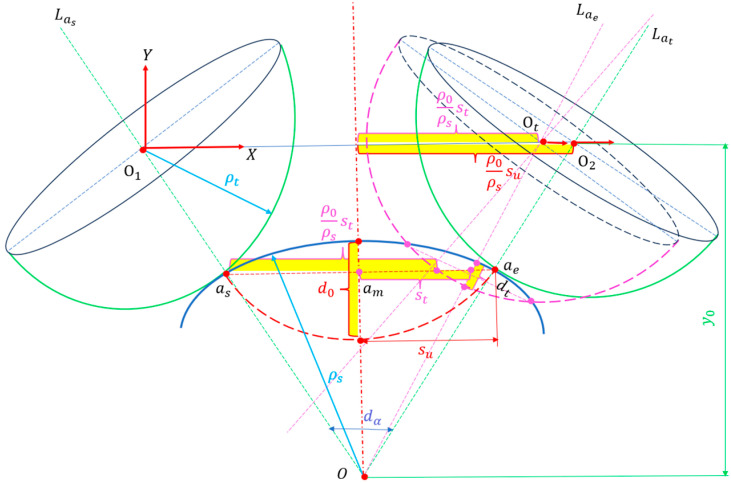
CFP diagram of a certain frequency band with the same workpiece curvature radius.

In any spatial frequency band, any $a_{s}a_{e}$ band with the same curvature radius $\rho_{s}$ is intercepted, and it is assumed that during the period from point $a_{s}$ to point $a_{e}$, the trajectory of the polishing tool’s rotation center $O_{1}$ to $O_{2}$ is a straight line, the total accumulated vertical deformation is $d_{0}$, and the total contact displacement is $s_{u}$. The center point of any position is $O_{t}$, located on the straight line $O_{1}O_{2}$, and its right contact displacement is $s_{t}$. The principle of CFP in a certain frequency band is shown in Figure 5.

The geometry of the CFP during the $a_{s}a_{e}$ band is as follows:

(13) $${s_{u}\left(d_{0}\right)}^{2}=\left(2\rho_{0}d_{0}-{d_{0}}^{2}\right)\cdot {\left(\rho_{s}∕\rho_{0}\right)}^{2}$$

Formula (13) is substituted into Formula (2), and the geometric relationship between the removal function and the spatial wavelength is established. According to the total contact displacement, $s_{u}$ is approximately the spatial wavelength $\Lambda_{0}$ of the frequency band, and the full width at half maximum (FWHM) is $\Lambda_{h}$; thus, the distribution formulas of variable-curvature factor ξ and variable-curvature pressure can be obtained, as shown in the following expressions:

(14) $${\xi =e}^{\frac{{\left(\pi \cdot \Lambda_{h}\cdot \Lambda_{0}\right)}^{2}}{\ln2\cdot \left(2\Lambda_{0}-1\right)}}$$

(15) $$\sigma_{t}\left(x_{t},y_{t},d_{t}\right)=\frac{k_{t}{\xi E}_{1}{\cdot \left(2{\rho_{t}d}_{te}-{d_{te}}^{2}\right)}^{3∕2}\cdot {\left({c_{t}(d_{t})}^{2}-{x_{t}}^{2}-{y_{t}}^{2}\right)}^{1/2}}{\rho_{t}\cdot {c_{t}(d_{t})}^{3}}$$

### 2.4. Simulation of Pressure Distribution on Surface with Variable Curvature

**Figure 6:**
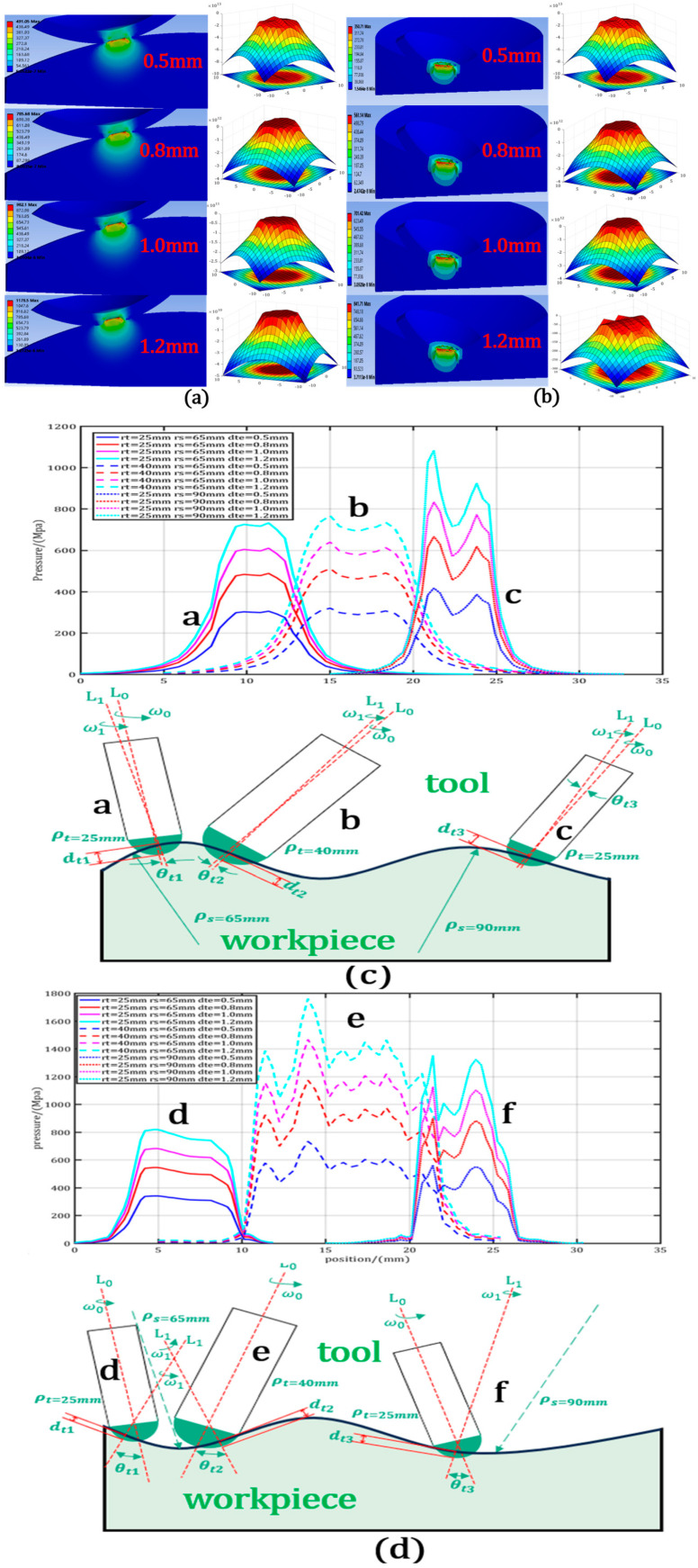
Simulation of pressure distribution on surfaces with variable curvature. (**a**,**b**) Pressure simulation results of convex and concave workpieces; (**c**,**d**) changes in pressure distribution of convex and concave workpieces with change in the workpiece curvature radius. $\rho_{s}$ (65 and 90 mm) and tool curvature radius $\rho_{t}$ (25 and 40 mm).

In order to study the pressure distribution of a variable-curvature workpiece and the removal of material by the CFP tool, a finite element analysis (FEA) was performed using Ansys 16.0 software. According to the variable-curvature pressure distribution Equation (14), the pressure decreases as the contact radius increases. By modifying the offset, local coupling between the two surfaces was achieved, creating a series of contact pairs. The material properties used for the model are detailed in Table 1. Therefore, the constrained displacement of the bottom surface of the workpiece was set to 0 mm, the removal amount on the x- and y-axes was set to zero, and the displacement amount on the Z-axis was set to the conditions of −0.5 mm, −0.8 mm, −1 mm, and −1.2 mm, respectively, to study the influence of the pressure distribution on the characteristics of the convex and concave surfaces, as shown in Figure 6a,b.

As shown in Figure 6c, it can clearly be seen from the preliminary simulation results of the pressure distribution of the convex workpiece that when the curvature radius of the workpiece is the same, the peak pressure gradually increases, but the FWHM change is small, and the pressure distribution has a gradual depression in the middle like an “M”. When the workpiece curvature radius is unchanged, as the tool curvature radius increases, the FWHM gradually increases, but the pressure change in the middle is relatively small. As shown in Figure 6d, it is evident from the preliminary simulation results of the pressure distribution of the concave workpiece that when the curvature radius of the workpiece is the same, the peak pressure increases slightly, the pressure distribution diverges gradually, and the consistency weakens. Compared with the convex workpiece, the pressure becomes flatter, and the FWHM also increases. When the curvature radius of the workpiece is unchanged, with the increase in the curvature radius of the tool, the FWHM increases greatly. Although the pressure distribution gradually diverges, the change amplitude is small. With the increase in the downward pressure, there are abnormal extreme points at random positions.

When the material properties of the workpiece and the tool are set to a certain extent, the curvature radius $\rho_{s}$ of the optical element, the tool radius of curvature $\rho_{t}$, and the actual offset $d_{t_{e}}$ are used as variable factors. Taking the total vertical deformation of the workpiece and tool $d_{t}$, contact radius of workpiece and tool $c_{t}$, contact angle of workpiece and tool $\theta_{t}$, and maximum contact pressure $\sigma_{t}$ as response indexes, a total of 16 groups of different structural parameters were used to establish pressure simulation experiments on convex and concave workpieces. The results are shown in Table 2 and Table 3, respectively.

It can be seen from the data in the table that for convex workpieces, the total deformation $d_{t}$ and contact radius $c_{t}$ are more sensitive to the tool curvature radius than the workpiece curvature radius, and they increase with the increase in downward pressure. However, the initial radius decreases with the increase in tool curvature radius and workpiece curvature radius and is independent of the amount of downward pressure. The edge contact angle increases with the increase in the lower pressure and is sensitive to the tool curvature radius when the lower pressure is small and to the workpiece curvature radius when the lower pressure is large. For concave workpieces, the total deformation and initial radius increase with the increase in tool curvature radius and decrease with the increase in workpiece curvature radius; the latter is independent of the downward pressure, and the contact radius is more sensitive to the workpiece curvature radius than the tool curvature radius and increases with the increase in downward pressure. The edge contact angle increases with the increase in the pressure, decreases with the decrease in the workpiece and tool curvature radius, and is more sensitive to the latter.

## 3. Experimental

### 3.1. Experimental Procedure

According to the simulation results of variable-curvature pressure above, the concave–convex characteristics of the workpiece have a great influence on the distribution of compressive stress, that is, the weight of the surface of the workpiece with variable curvature has a greater influence on the distribution of the removal function. Therefore, experiments are needed to deeply verify the matching relationship between the figuring tool and the workpiece, that is, the fit degree between the FWHM of the removal function obtained by the modification test with the spatial wavelength of the machined surface. Therefore, in order to achieve full-size freeform figuring, it is necessary to generate a series of stable TIFs, so that the figuring tool can adapt to the spatial wavelength of each band as it passes through the entire surface. In our previous pre-experiment [19], L16 orthogonal experiments and analysis of variance (ANOVA) methods were conducted on flat specimen surfaces on a 100 mm diameter Ni–P plate in an ultra-smooth modification test machine (CFP1000, Harbin, China), utilizing a sponge head as the figuring tool.

#### 3.1.1. Conver TIFs

**Figure 7:**
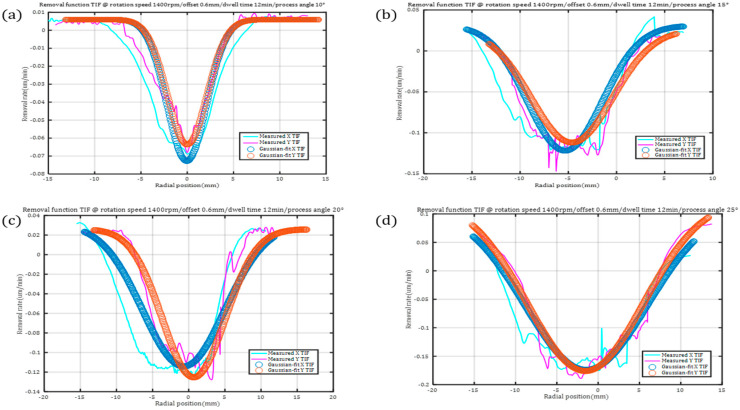
Different removal functions of four TIFs with measured and Gaussian fit in conditions of rotation speed 1400 rpm, offset 0.6 mm, dwell time 12 min, and process angles 10°, 15°, 20°, and 25°, respectively.

According to Formula (14) and the simulation results of variable-curvature pressure, the TIF of the plane was converted into the TIF of the surface and fitted as shown in Figure 7.

When the process angle is reduced to 10°, the FWHM of the fitted removal function is reduced to a Gaussian-like shape, and the removal resolution improves, but the removal efficiency is reduced, and the amplitude of the dwell time is obviously increased.

#### 3.1.2. Fit of the Removal Function

The removal function generated by fitting is uni-Gaussian (*TIF2*), multi-Gaussian (*TIF3*), and cosinized (*TIF4*), and the expressions are as follows:

(16) $$TIF1=-0.074\cdot e^{-\frac{4\ln2\cdot x^{2}}{6.57^{2}}}$$

(17) $$TIF2=-0.12\cdot e^{-\frac{4\ln2\cdot x^{2}}{5.06^{2}}}-0.05\cdot e^{-\frac{4\ln2\cdot x^{2}}{3.66^{2}}}-0.04\cdot e^{-\frac{4\ln2\cdot x^{2}}{3.41^{2}}}$$

(18) $$TIF3=0.005\cdot e^{-\frac{4\ln2\cdot x^{2}}{19.90^{2}}}-0.15\cdot e^{-\frac{4\ln2\cdot x^{2}}{13.34^{2}}}-0.03\cdot e^{-\frac{4\ln2\cdot x^{2}}{99^{2}}}$$

(19) $$TIF4=-0.06-0.1\cdot \cos\left(\frac{x}{1.6\pi }\right)+0.05\cdot \sin\left(\frac{x}{1.6\pi }\right)$$

#### 3.1.3. Different-Order Filtration of TIF

**Figure 8:**
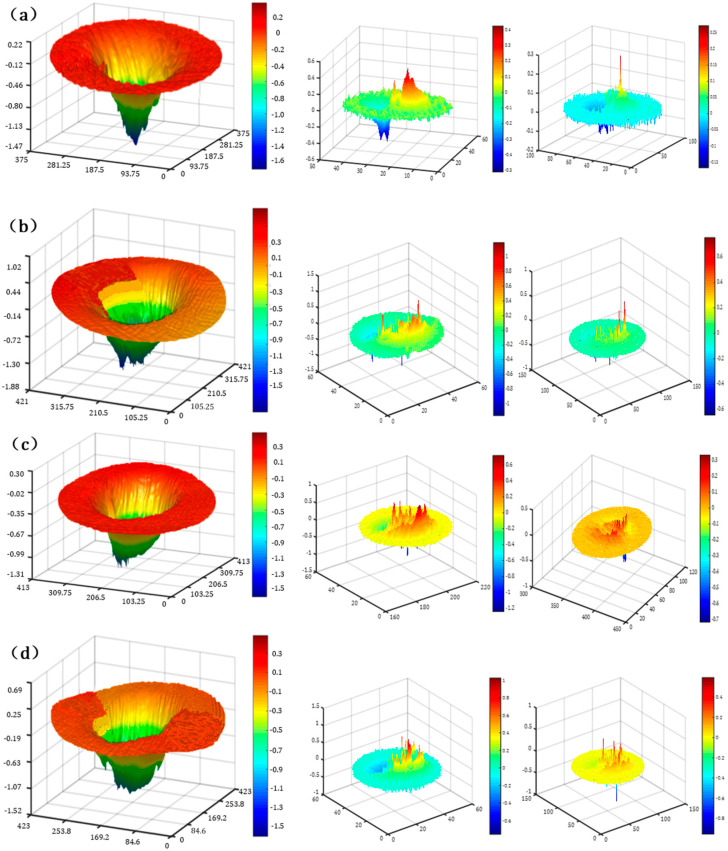
Four TIFs with low-, medium-, and high-order filtering for variable-curvature polishing. (**a**) *TIF1*; (**b**) *TIF2*; (**c**) *TIF3*; (**d**) *TIF4*.

Low-, medium-, and high-order filtration of the TIF on variable-curvature surfaces was carried out for the selection of a suitable TIF, as shown in Figure 8. The removal function was filtered by frequency division, the processing error of the high-frequency band was removed after the low-order frequency filtering, and the removal function contour was obtained. After the middle-order frequency filtering, the maximum removal depth of the middle fluctuated greatly, which proves that there is an obvious peak in the corresponding PSD curve. After high-order frequency filtering, the removal function was evenly distributed, and the fluctuation was small and stable, which proves that the PSD curve and coordinate axis in the high frequency band are smaller in area and amplitude, and the surface roughness of high frequency is further improved. Therefore, on the whole, the low-order profile of the removal function is stable for the Gaussian-like type, the removal function has small fluctuations in the middle level, and the removal function has small, stable fluctuations in the higher order, which proves that the removal function is stable in unit time.

#### 3.1.4. The 1D-PSD Simulation

**Figure 9:**
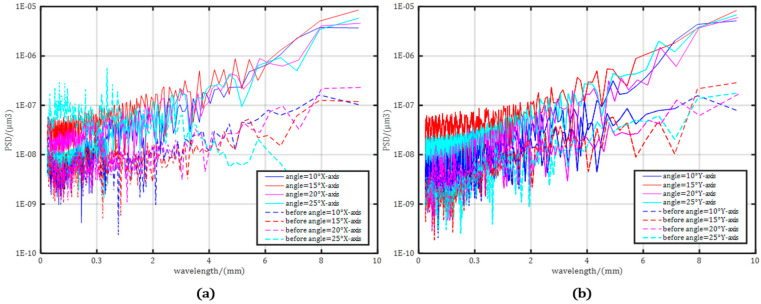
PSD results of four TIFs: (**a**) a convex part; (**b**) a concave part.

The power density spectrum (PDS) of the surface roughness shows the change in surface deviation with the length of the trajectory, i.e., the spatial period. The actual PSD curve can be approximated within a defined region by two adjacent spatial frequencies, while representing roughness changes at different locations on the same surface. The solid line represents PSD without figuring, and the dotted line represents the PSD after figuring. The logarithmic plot of the analysis results of PSD and wavelength is shown in Figure 9, where the slope of the straight line is different at different wavelengths, and the primary gradient of 1D-PSD is approximately the same on the basis of the same optical sample. At the same time, the straight lines with the same slope of translation in different 1D-PSD bands can characterize the PSD peaks and PSD trends under different RMS values. The increase in the bounding area of the coordinate axis and the contouring of the PSD curve will lead to an increase in the RMS wavefront error, but the PSD peak value is significantly suppressed in a specific wavelength range.

#### 3.1.5. Dwell Time Calculation for Variant TIFs

**Figure 10:**
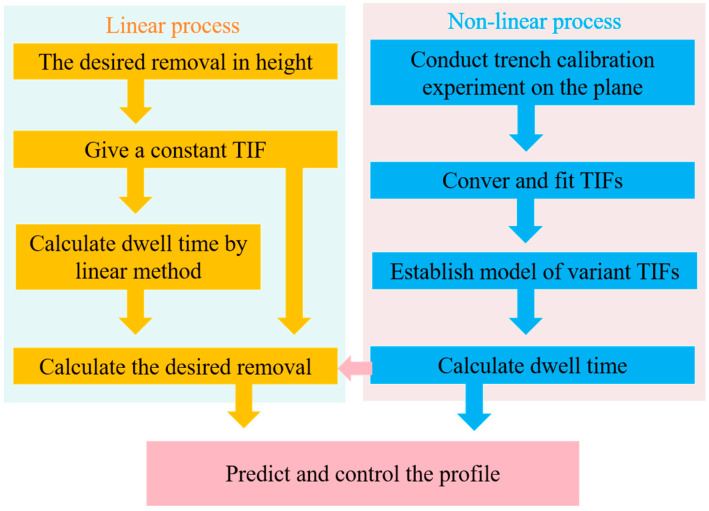
Flowchart of the nonlinear method for dwell time calculation.

Generally, the linear process can be regarded as the freeform surface generation process using a constant TIF [20], in which the desired removal is determined by the linear deconvolution calculation, as shown in Figure 10. On the other hand, the nonlinear process uses variant TIFs, in which the relationship between removal and dwell time is obtained by trench calibration experiments on the plane. In this way, the calculation method establishes the model of variant TIFs by considering the nonlinear effect of variant TIFs and also avoids complex mathematical calculation.

### 3.2. Variable-Curvature Mandrel-Figuring Experiment

**Figure 11:**
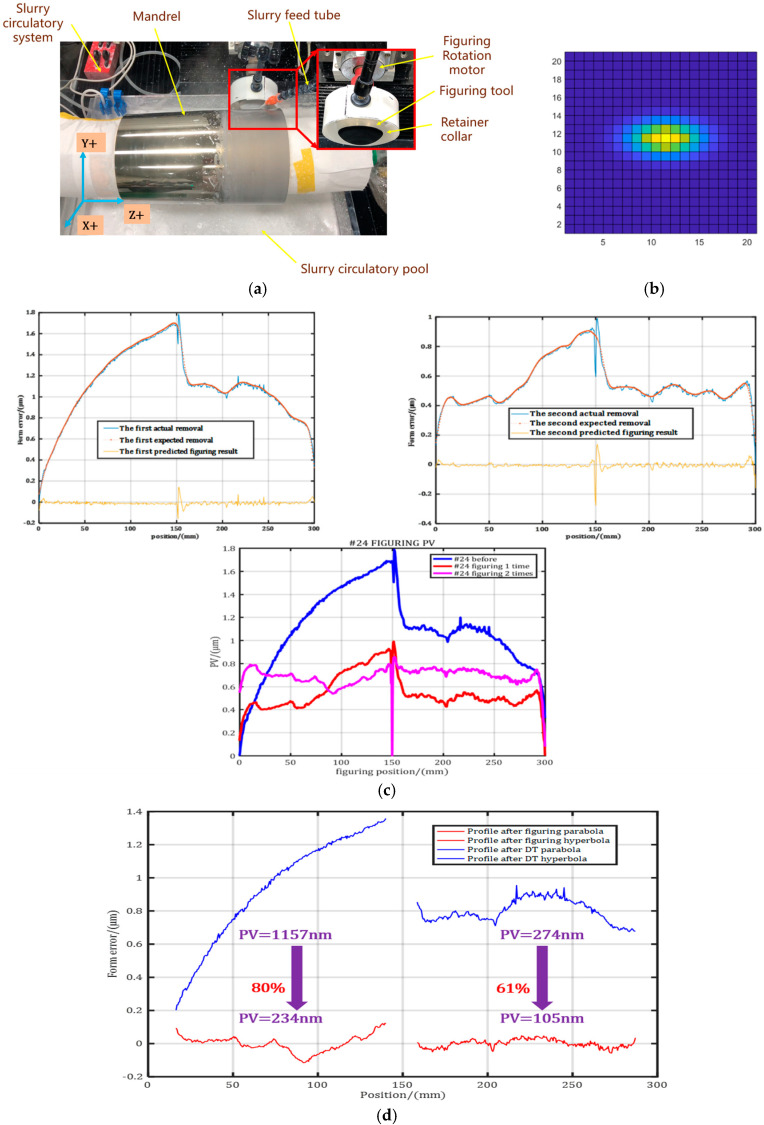
The mandrel figuring experiment. (**a**) Principle of figuring variable-curvature mandrel on CFP1000. (**b**) An ideal initial elliptic Gaussian removal function. (**c**) Initial profile of #24 mandrel and twice figuring with calculation and prediction. (**d**) Profile accuracy improving results for paraboloid and hyperboloid.

The continuous flexible modification method is used to install large freeform surface optical devices on the ultra-smooth modification test machine CFP1000 (HIT, Harbin, China) to achieve rapid convergence of optical surface accuracy while maintaining high surface roughness. A slurry with a concentration of 40 g/L containing a 20 nm silica suspension and pure water is supplied in real time through a feed slurry tube connected to the peristaltic pump to the flexible finishing head and delivered to the edge of the finishing area. At the same time, a slurry concentration of 20 g/L is provided through the circulation system and spray choke to ensure that stability is supplemented, and the abrasive particles are gradually carried by the starch structure from the tool edge to the molding area. This technique avoids the uneven spread of abrasive particles in the early modification phase, guaranteeing consistent material removal [21]. To avert surface damage caused by the drying and crystallization of untreated areas, a fine mist of pure water is applied to keep the submicron layer of the optical surface highly humid. The experimental setup involved a 40 mm radius flexible tool and a 20-degree precession angle. The spindle of the modification tester rotated at a constant speed of 20 rpm, and the modification tool moved from right to left in the range of 300 rpm to 1500 rpm to modify the mandrel using a spiral trajectory, as shown in Figure 11a. According to the four TIF characteristics in Section 3.1, a personalized variable-curvature polishing TIF can be obtained by calibrating an ideal initial elliptic Gaussian removal function, as shown in Figure 11b. The residence time was calculated after the removal of the kernel, and the use of residence time as a basis for control provided a predictable process, as shown in Figure 11c. Meanwhile, the contour surface shape in the initial state presented irregular high and low undulation measured by the STIL spectral confocal sensor, and the mandrels’ profile accuracy metrology was introduced [10]. The multi-step flexible figuring iteration was carried out for the rotating symmetric variable-curvature mandrel using a time-varying deconvolution algorithm. As shown in Figure 11d, the experimental results show that the shape accuracy of the parabolic surface is reduced from 1157 nm PV to 234 nm PV, and that of the hyperbolic surface is reduced from 274 nm PV to 105 nm PV.

### 3.3. The Surface Quality and the Optical Performance of a Mirror

To comprehensively investigate the effect of CFP on the ripple of the MSF error, sub-aperture iterative figuring for the variable-curvature mandrel was conducted utilizing the experimental parameters in Table 4.

**Figure 12:**
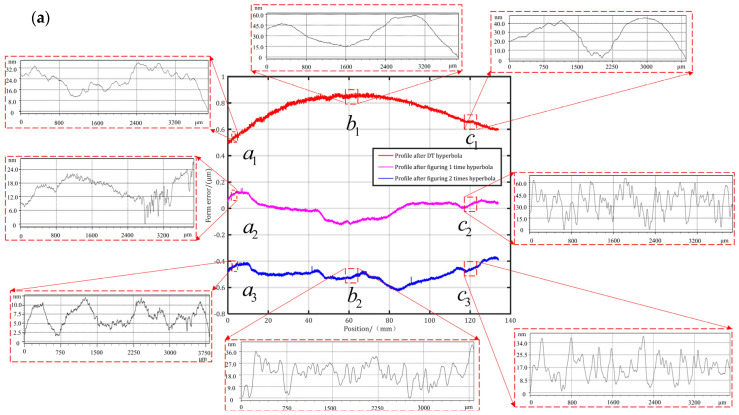

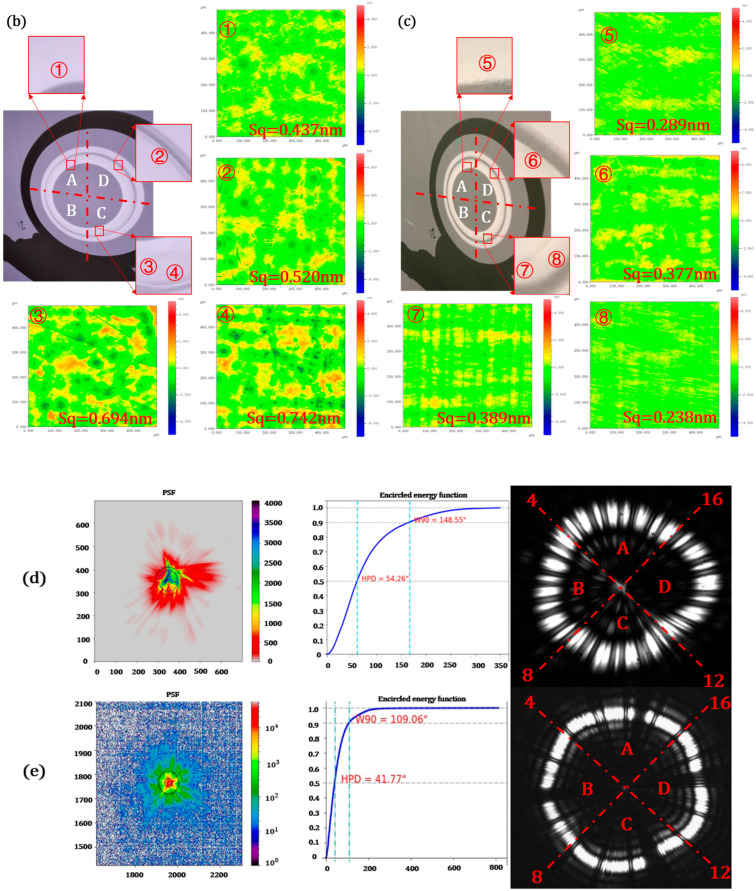
The iterative figuring of the variable-curvature optical surface: (**a**) The enhancement of the form accuracy; (**b**) the improvement of surface roughness with the star-point method; (**c**) the promotion of the optical performance with the visible light observation approach; (**d**) the PSF image and the encircled energy curve before iterative figuring; (**e**) the PSF image and the encircled energy curve after iterative figuring.

The figured optical mold was employed to fabricate multilayer film optics to verify the figuring capability of CFP through the star-point approach [22] and the overhang optical element visible light observation approach [23]. The multilayer film optics verified with the star-point method at the middle curvature radius had good quality performance before the figuring process, which was further optimized following the iterative figuring. The surface roughness of the optical element at the corresponding position was improved from 0.437 nm RMS to 0.289 nm RMS. The imaging performance at the edge of the multilayer film optics was evidently improved and relieved the ghosting phenomenon after iterative figuring. The surface roughness at the corresponding position was enhanced from 0.52 nm RMS and 0.742 nm RMS to 0.377 nm RMS and 0.238 nm RMS, respectively. Furthermore, the imaging clutter was effectively weakened after iterative figuring in the part with the undersized curvature radius. The surface roughness at the corresponding position increased from 0.694 nm RMS to 0.389 nm RMS, as shown in Figure 12b,c.

Optical quality evaluation is a critical process in optical precision manufacturing. As depicted in Figure 12d,e, the multilayer film optics are overhung by the suspension wire connected with the attitude control device to obtain the PSF image and the encircled energy (EE) curve. A light source releases parallel light with a wavelength of 473 nm from below grazing incidence into the interior of the optic, and the CMOS camera, located above, captures the minimum spot of the optics imaging to evaluate the optical performance quality. The spot-imaging pattern is divided into four regions and sixteen directions for detailed analysis of the optical properties. Comparing the results of the spot imaging before and after the iteration, the small-angle scattering phenomenon of the PSF image is significantly suppressed in regions A, B, and C, and the large deformation in the D region is sharply reduced by the enhancement of the surface finish and the adjustment of the attitude device. The angular resolution depends on the optical die surface roughness and form accuracy. The SDPT and lapping polishing process control the LSF and HSF errors, while the CFP process controls the MSF error, as mentioned in Section 2.1. Consequently, the half-power diameter (HPD) and W90 are separately enhanced from 54.26 arcsec and 148.55 arcsec to 41.77 arcsec and 108.06 arcsec.

## 4. Conclusions

In this paper, time-varying flexibility and dwelling-time-based CFP optical surface correction approaches are proposed and investigated. The conclusions of this study can be summarized as follows:

1. Based on the variable-curvature characteristics and time-varying removal function of the parabolic–hyperbolic optical surface, a variable-curvature pressure model is established to derive the variable-curvature factor function. Additionally, the relationship between the variable-curvature surface and the plane is formulated by relating the actual downward pressure to key parameters, such as contact radius and contact angle.
2. Utilizing the residence time distribution of various TIF models, along with hierarchical filtering of TIF and PSD distribution, the variable-curvature surface and surface guide each other, allowing for real-time modification of TIF to achieve customized variable-curvature polishing.
3. Employing a time-varying deconvolution algorithm, multi-wheel flexible polishing iterations were performed on the rotationally symmetric mandrel with variable curvature. The experimental results demonstrate that the shape accuracy of the parabolic surface was improved from 1157 nm PV to 234 nm PV, while that of the hyperbolic surface was reduced from 274 nm PV to 105 nm PV. These results significantly reduce MSF ripple and enhance shape accuracy.
4. The optical quality evaluation with the visible light observation approach also verifies that the HPD and W90 were enhanced.

## Figures and Tables

**Table 1:** Simulation parameters of figuring process on Ni-P surface.

Material	Density (kg/m^3^)	Elastic Modulus (Pa)	Poisson’s Ratio
Compliant tool head	0.035 × 10^2^	9.0 × 10^8^	0.38
Ni-P alloy	0.090 × 10^2^	1.14 × 10^11^	0.35
Al alloy	0.277 × 10^4^	7.0 × 10^10^	0.33

**Table 2:** Simulation results of convex pressure.

Workpiece Type	Workpiece Curvature Radius $\rho_{s}/\left(mm\right)$	Tool Curvature Radius $\rho_{t}/\left(mm\right)$	Offset $d_{te}/\left(mm\right)$	Total Deformation $d_{t}/\left(mm\right)$	Contact Radius $c_{t}/\left(mm\right)$	Initial Contact Radius $c_{0}/\left(mm\right)$	Contact Angle $\theta_{t}/{(}^{\circ })$	Pressure $\sigma_{t}/\left(\mathrm{Mpa}\right)$
Convex	65	25	0.5	0.5132	5.800	5.8647	68.03	304.84
			0.8		0.8212	6.300	78.28	487.74
			1.0		1.0265	6.850	82.42	609.67
			1.2		1.2318	7.000	89.92	731.61
		40	0.5	0.5170	10.30	2.7130	70.82	319.49
			0.8		0.8272	10.64	74.58	511.19
			1.0		1.0340	11.41	76.49	638.99
			1.2		1.2408	12.00	78.28	766.79
	90	25	0.5	0.5129	5.070	3.7062	61.90	451.31
			0.8		0.8207	5.750	72.22	722.09
			1.0		1.0258	6.250	79.08	902.62
			1.2		1.2310	7.500	83.09	1083.00
		40	0.5	0.5176	9.877	4.9049	68.47	376.46
			0.8		0.8282	10.14	74.03	602.34
			1.0		1.0352	10.95	82.38	752.93
			1.2		1.2422	12.00	88.09	903.15

**Table 3:** Simulation results of concave pressure.

Workpiece Type	Workpiece Curvature Radius $\rho_{s}/\left(mm\right)$	Tool Curvature Radius $\rho_{t}/\left(mm\right)$	Offset $d_{te}/\left(mm\right)$	Total Deformation $d_{t}/\left(mm\right)$	Contact Radius $c_{t}/\left(mm\right)$	Initial Contact Radius $c_{0}/\left(mm\right)$	Contact Angle $\theta_{t}/{(}^{\circ })$	Pressure $\sigma_{t}/\left(\mathrm{Mpa}\right)$
Concave	−65	25	0.5	0.5165	4.8775	3.6642	73.29	340.79
			0.8		0.8263	5.2265	76.41	545.25
			1.0		1.0329	5.5245	78.31	681.57
			1.2		1.2395	5.9275	80.61	817.89
		40	0.5	0.5193	6.8555	6.1996	28.12	700.00
			0.8		0.8309	6.9534	38.33	1000.00
			1.0		1.0386	7.2504	47.51	1250.00
			1.2		1.2463	7.4554	53.24	1500.19
	−90	25	0.5	0.5148	7.2650	3.0039	71.10	563.02
			0.8		0.8237	7.4000	71.94	900.84
			1.0		1.0296	7.5500	72.76	1126.00
			1.2		1.2355	7.6890	73.49	1351.30
		40	0.5	0.5216	8.1473	5.6370	75,54	668.57
			0.8		0.8346	8.9621	68.08	1069.70
			1.0		1.0432	9.3699	57.30	1337.10
			1.2		1.2518	9.7780	85.26	1604.60

**Table 4:** Experimental parameters of CFP figuring ability analysis.

Trial	FWHM (mm)	Removal Rate (mm^3^/min)	Spatial Length (mm)	PV (μm)
1	23, 17.5, 7.5	0.0202, 0.013, 0.012	30	1.230, 0.587, 0.520
2	20.5	0.0200	30, 40, 60	1.254, 1.263, 1.283
3	15.4	0.0385	20, 30, 60, 80	0.602, 0.607, 0.584, 0.599
4	8.5	0.0120	20, 30, 60, 80	0.550, 0.527, 0.519, 0.542

## Data Availability

The original contributions presented in this study are included in the article. Further inquiries can be directed to the corresponding authors.

## References

[1] Martin H.M., Allen R.G., Burge J.H., Kim D.W., Kingsley J.S., Tuell M.T., West S.C., Zhao C., Zobrist T. (2010). Fabrication and testing of the first 8.4-m off-axis segment for the giant magellan telescope. Modern Technologies in Space-and Ground-Based Telescopes and Instrumentation.

[2] Zhang Y., Wang Y., Wang M., Guo Y., Li X., Chen Y., Lu Z., Wu J., Ji X., Dai Q. (2022). Multi-focus light-field microscopy for high-speed large-volume imaging. PhotoniX.

[3] Huang B., Li J., Yao B., Yang Z., Lam E.Y., Zhang J., Yan W., Qu J. (2023). Enhancing image resolution of confocal fluorescence microscopy with deep learning. PhotoniX.

[4] Citterio O., Bonelli G., Conti G., Mattaini E., Santambrogio E., Sacco B., Lanzara E., Brauninger H., Burkert W. (1988). Optics for the x-ray imaging concentrators aboard the x-ray astronomy satellite SAX. Appl. Opt..

[5] Brinkman A.C., Behar E., Güdel M., Audard M., den Boggende A.J.F., Branduardi-Raymont G., Cottam J., Erd C., den Herder J.W., Jansen F. (2001). First Light Measurements with the XMM-Newton Reflction Grating Spectrometers: Evidence for an Inverse First Ionisation Potential Effect and Anomalous Ne A bundance in the Coronae of HR 1099. Astron. Astrophys..

[6] Citterio O., Campana S., Conconi P., Ghigo M., Mazzoleni F., Poretti E., Conti G., Cusumano G., Sacco B., Braeuninger H.W. (1996). Characteristics of the flight model optics for the JET-X telescope onboard the Spectrum-X-Gamma satellite. Proceedings of the Multilayer and Grazing Incidence X-Ray/EUV Optics III.

[7] Weisskopf M.C., Ramsey B., O’dell S.L., Tennant A., Elsner R., Soffita P., Bellazzini R., Costa E., Kolodziejczak J., Kaspi V. (2016). The Imaging X-ray Polarimetry Explorer (IXPE). Results Phys..

[8] Predehl P., Andritschke R., Arefiv V., Babyshkin V., Batanov O., Becker W., Böhringer H., Bogomolov A., Boller T., Borm K. (2020). The eROSITA X-ray telescope on SRG. Astron. Astrophys..

[9] Friedrich P., Stieglitz V., Burwitz V., Eder J., Dennerl K., Hartner G., Langmeier A., Müller T., Rukdee S., Schmidt T. (2024). X-ray optics test and calibration of the Einstein Probe Follow-up telescope. Acta Astronaut..

[10] Wolter H. (1952). Glancing incidence mirror systems as imaging optics for X-rays. Ann. Physik.

[11] Liao Q., Ding F., Chen Z., Li D., Wang B. (2023). Study on the Fabrication Process of X-ray Focusing Mirrors. Micromachines.

[12] Yamaguchi G., Matsuzawa Y., Kume T., Imamura Y., Miyashita H., Ito A., Sakuta K., Ampuku K., Fujii R., Hiraguri K. (2023). Efficient and precise fabrication of Wolter type-I x-ray mirrors via nickel electroforming replication using quartz glass mandrels. Rev. Sci. Instrum..

[13] Xue J., Wang B., Liao Q., Wu K., Liu Y., Wu Y., Chen W., Qiao Z., Jin Y., Ding F. (2024). Precision Manufacturing in China of Replication Mandrels for Ni-Based Monolithic Wolter-I X-ray Mirror Mandrels. Aerospace.

[14] Egawa S., Owada S., Motoyama H., Yamaguchi G., Matsuzawa Y., Kume T., Kubota Y., Tono K., Yabashi M., Ohashi H. (2019). Full-field microscope with twin Wolter mirrors for soft X-ray free-electron lasers. Opt. Express.

[15] Vernani D. (2011). Advanced Manufacturing Techniques for X-Ray and VHE Gamma-Ray Astronomical Mirrors. Ph.D. Thesis.

[16] Kilaru K., Ramsey B.D., Baumgartner W.H., Bongiorno S.D., Broadway D.M., Champey P.R., Davis J.M., O’dell S.L., Elsner R.F., Gaskin J.A. (2019). Full-shell x-ray optics development at NASA Marshall Space Flight Center. J. Astron. Telesc. Instrum. Syst..

[17] Preston F. (1927). The theory and design of plate glass polishing machines. J. Glass Technol..

[18] Schinhaerl M., Rascher R., Stamp R., Smith L., Smith G., Sperber P., Pitschke E. (2008). Utilisation of time-variant influence functions in the computer controlled polishing. Precis. Eng..

[19] Wan S., Zhang X., Zhang H., Xu M., Jiang X. (2018). Modeling and analysis of sub-aperture tool influence functions for polishing curved surfaces. Precis. Eng..

[20] Ren L., Zhang G., Zhang L., Zhang Z., Huang Y. (2019). Modelling and investigation of material removal profile for computer controlled ultra-precision polishing. Precis. Eng..

[21] Zhang L., Tam H.Y., Yuan C.-M., Chen Y.-P., Zhou Z.-D. (2002). An investigation of material removal in polishing with fixed abrasives. Proc. Inst. Mech. Eng. Part B J. Eng. Manuf..

[22] Li Y., Li G., Xue J., Ding F., Wang B. (2024). Mechanism of a deterministic sponge figuring processing (SFP) in Ni–P surface formation considering the robustness of the tool influence function: Modeling and experimental investigation. Int. J. Adv. Manuf. Technol..

[23] Yu K., Li F., Yang Y., Zhao Z., Lu B., Zhang J., Chen Y., Wu K. (2023). Analysis of Diffraction Effects in Visible Wavelength Tests for Wolter-I X-Ray Telescope. Laser Optoelectron. Prog..

